# Non-ketotic Hyperglycemic Hemichorea-Hemiballismus: A Case of a Male With Diabetes Mellitus and Speech Disturbances

**DOI:** 10.7759/cureus.25073

**Published:** 2022-05-17

**Authors:** Catherine M Evers Smith, Kajol K Chaurasia, Daniel C Dekoski

**Affiliations:** 1 Department of Neurology, Wright State University Boonshoft School of Medicine, Dayton, USA; 2 Department of Internal Medicine, Wright State University Boonshoft School of Medicine, Dayton, USA

**Keywords:** hyperintense basal ganglia, dysarthria, diabetes mellitus, hyperglycemic hemichorea, hemichorea-hemiballismus

## Abstract

Non-ketotic hyperglycemic chorea-ballismus is characterized by a triad of chorea-ballismus, non-ketotic hyperglycemic state, and striatopathy on magnetic resonance imaging (MRI) or computerized tomography scan (CT). Chorea and ballismus are hyperkinetic movements affecting the side contralateral to the striatal hyperintensity on imaging. This presentation is a manifestation of poorly controlled diabetes mellitus most commonly reported in elderly eastern Asian women. The exact mechanism is unknown, but it is reversible with optimal glycemic control. The patient described in this case is a Caucasian male in his mid-50s who presented to the emergency department with speech disturbance. Only after a detailed neurologic examination and MRI head in the setting of non-ketotic hyperglycemia the diagnosis of non-ketotic hyperglycemia hemichorea-hemiballismus was deduced. Consideration of this disease in patients with poorly controlled diabetes mellitus is important as tight glycemic control can be implicated to reverse the condition.

## Introduction

Non-ketotic hyperglycemic chorea-ballismus, also known as chorea hyperglycemia basal ganglia syndrome, is a rare manifestation of poorly controlled diabetes mellitus most commonly reported in women of eastern Asian descent with a mean age of 71 years [1]. It is characterized by a triad of acute or subacute chorea-ballismus, non-ketotic hyperglycemic state, and hyperintense striatopathy on magnetic resonance imaging (MRI) or computerized tomography scan (CT), most commonly the putamen [2]. Hemichorea and hemiballismus are hyperkinetic movements that affect the contralateral side to striatal hyperintensity on neuroimaging. These MRI features have also been described in cases of hepatic encephalopathy, postcardiac arrest encephalopathy, long-term parenteral nutrition, and hypoglycemic-induced coma [3].

Although the exact mechanism of non-ketotic hyperglycemic chorea-ballismus is unknown, there are a few hypothesized mechanisms [4]. The first is that hyperviscosity secondary to hyperglycemia causes disruption in the blood-brain barrier and regional metabolic failure. The second is that the increased sensitivity of dopamine receptors in the post-menopausal state leads to hyperkinesis. This proposed mechanism may explain the higher incidence in females. The third hypothesized mechanism is that the non-ketotic state decreases the availability of acetoacetate for conversion to gamma-aminobutyric acid (GABA) and thus limits the amount of GABA (the chief inhibitory neurotransmitter in the central nervous system) in the thalami/striatum [4]. Non-ketotic hyperglycemic chorea-ballismus is reversible by treatment with optimal glycemic control [2-6]. Most patients recover within days to months.

## Case presentation

A 55-year-old male presented to the emergency department with complaints of speech disturbances for one week. He had a past medical history of poorly controlled type 2 diabetes mellitus, left ischemic thalamic stroke three years ago, subarachnoid hemorrhage from a bicycle accident six months ago with residual left-sided weakness, hypertension, hyperlipidemia, schizoaffective disorder, and alcohol abuse. He had not been taking any antipsychotic medications. For the last five months, he has been experiencing frequent dizzy spells and falls, culminating in him striking his head a week prior to the presentation. He has also been experiencing frequent, unpredictable, and non-suppressible movements of the left side of his body. He had not previously sought medical care for these issues. The patient is historically non-adherent with his medical care.

Upon initial examination, he was noted to have fluctuating dysarthria and word-finding difficulty. He had a baseline deficit of pure motor left hemiparesis. Physical examination was notable for frequent, non-suppressible, unpredictable movements of his left face, arm, and leg. These movements were mainly low amplitude with random intermittent high amplitude movements. The hyperkinetic movements occurred at rest, posture, and action. The movements were not rhythmic. The movements of the leg, face, and arm were not synchronized and mainly involved the proximal muscles. The movements did not resolve or improve over the course of the patient’s two-day hospital stay. He was unable to identify when these movements began but endorsed falls over the past five months that had increased in frequency over the past week. He denied any periods of confusion or lethargy after these falls and any preceding auras. 

**Figure 1:**
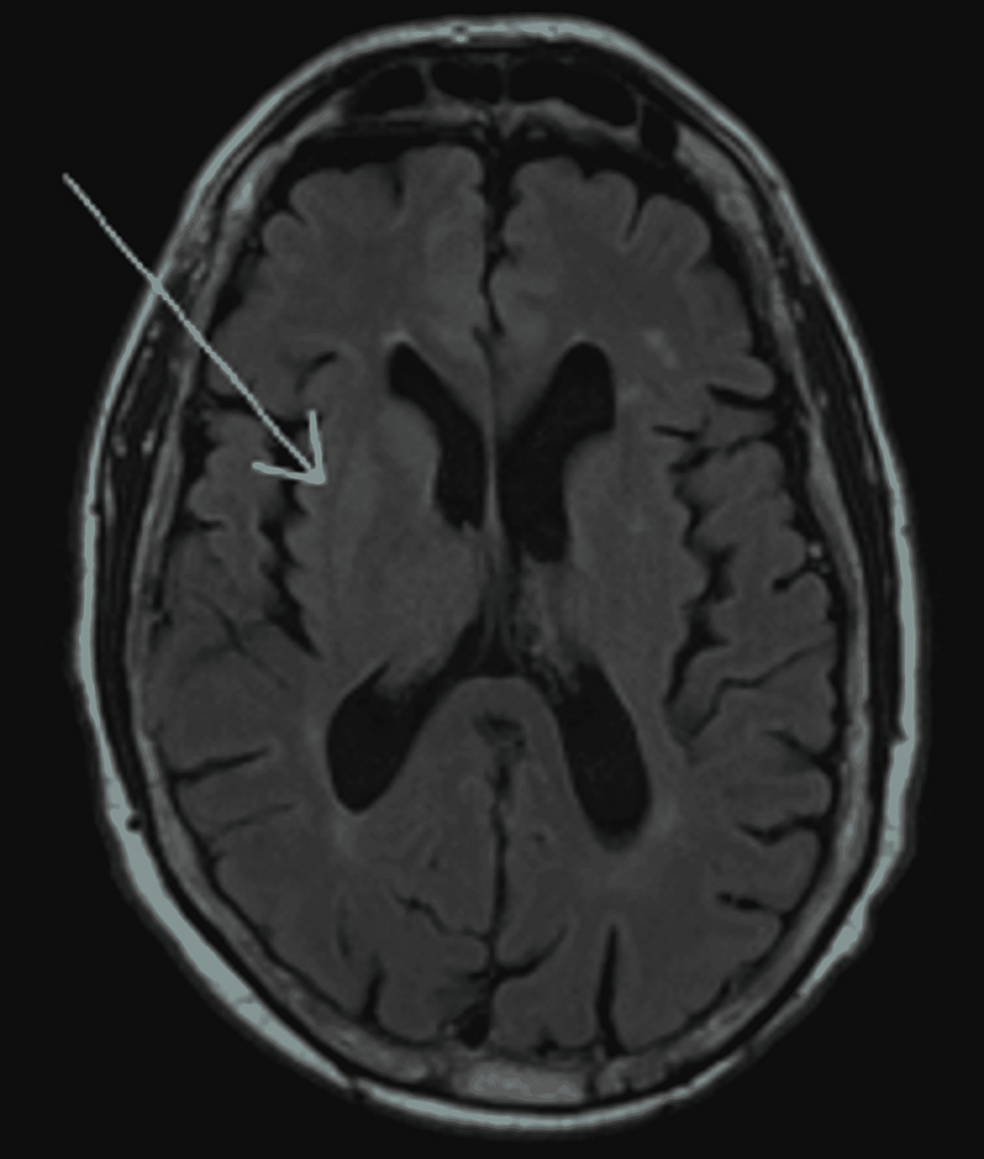
Axial view of non-enhancing T1 hyperintensity within the right putamen

**Figure 2:**
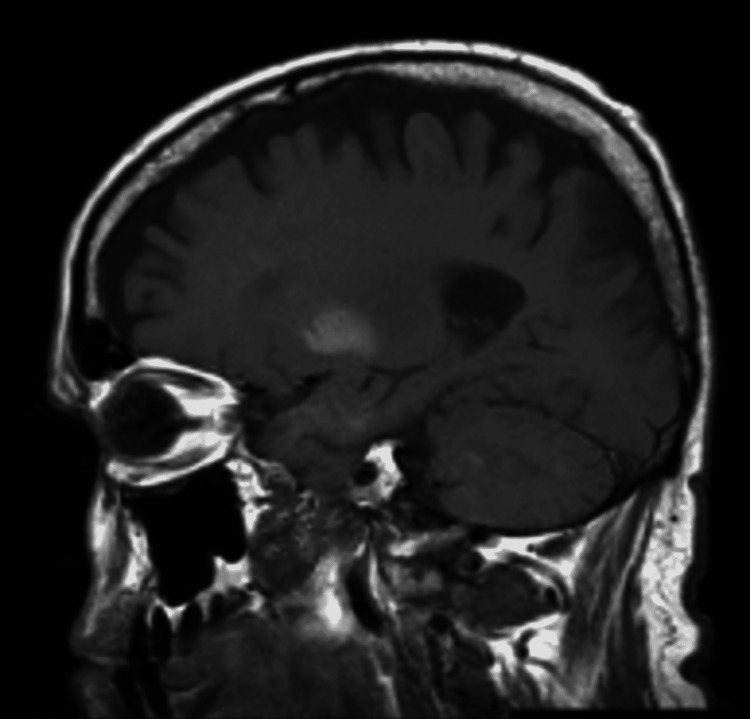
Sagittal view of non-enhancing T1 hyperintensity within right putamen

He had halting, non-fluent speech without paraphasic errors and mild dysarthria. He could name common objects and read short phrases. He was able to follow commands and recall one of three objects after two minutes. Cranial nerves II-XII were intact. Strength and primary sensory modalities were intact. He had difficulty relaxing his extremities, but deep tendon reflexes were 2/4 throughout. No clonus was elicited, and he had an absent Babinski. Computerized tomography head and cervical spine were without acute changes. MRI brain with and without contrast demonstrated an asymmetric non-enhancing lesion within the right putamen with low T2 and high T1 intensity subcortical signal (Figures 1, 2). 

These imaging findings were new from imaging three years ago. Labs were notable for blood glucose of 286 mg/dL, with previous records >500 mg/dL, and hemoglobin A1c of 12.4%. Treatment of this patient focused on aggressive blood glucose control with insulin, with recommendations to continue speech therapy and swallow study to assist in his ongoing dysarthria evaluation.

## Discussion

The patient in this vignette presented with dysarthria and hemiballismus. Dysarthria can result from injury to the upper or lower motor neuron systems, cerebellum, or extrapyramidal system. Hemiballismus narrows the localization to the basal ganglia, particularly the posterolateral putamen. This type of injury most commonly is a result of infarction, infection, neurodegenerative disorders, carbon monoxide poisoning, or neoplasm. Infarction is more likely to cause hemichorea than non-ketotic hyperglycemia. Ischemia was not seen on diffusion-weighted imaging/apparent diffusion coefficient (DWI/ADC) MRI sequences in this patient, making acute stroke unlikely. Infection leading to hemiballismus is typically due to cerebral toxoplasmosis in HIV-positive patients, which is not germane to this discussion. Neurodegenerative disorders such as Huntington's disease can lead to chorea of less acute onset. Huntington's disease typically has an earlier onset with generalized chorea. In Parkinson's disease, chorea can be observed as levodopa-induced dyskinesia neurological manifestations from carbon monoxide poisoning may occur 14-45 days post-acute exposure with generalized chorea. Neoplasm can have various clinical presentations with a mass seen on imaging. Hepatic encephalopathy must be included in the differential diagnosis to the patient’s history of alcohol abuse, however, the unremarkable liver panel argues against this. Seizures can occur in the setting of non-ketotic hyperglycemia and can also show basal ganglia hyperattenuation. However, the timeline in this patient and the lack of a post-ictal state argue against this. 

The patient exhibited the triad of acute or subacute chorea-ballismus, non-ketotic hyperglycemic state, and hyperintense striatopathy on neuroimaging. Other neurological abnormalities can accompany non-ketotic hyperglycemic states such as seizures, delirium, aphasia, nystagmus, hemiparesis, hemisensory loss, hemianopia, and coma [1]. Here, the patient presented with mild left-sided choreiform movements affecting the face, arm, and leg, with ballismus worse in the leg. Additionally, he had speech disturbances characterized by halting, non-fluent speech without paraphasic errors, and gradually resolving dysarthria since admission. Non-ketotic hyperglycemic hemichorea-hemiballismus is reversible by treatment with optimal glycemic control. Most patients recover within days to months, but T1-weighted MRI changes in the basal ganglia may persist despite clinical improvement for up to six months [5].

The patient’s odd speech disturbance prompted his presentation to the emergency department. The presenting dysarthria at first glance could have been attributed to residual effects of the patient’s prior left thalamic stroke along with alcohol abuse. Only after careful neurological examination and MRI brain, the diagnosis of non-ketotic hyperglycemic hemichorea-hemiballismus was determined. 

## Conclusions

Non-ketotic hyperglycemia chorea-ballismus is a rare but important consideration in patients with poorly controlled diabetes mellitus. Prognosis is favorable with reversibility of chorea-ballismus and prevention of further neurological complications with adequate glycemic control.
